# RelA and mitogen-activated protein kinase kinase kinases potently enhance lentiviral vector production

**DOI:** 10.1016/j.bbrep.2024.101637

**Published:** 2024-02-01

**Authors:** Shoji Yamaoka

**Affiliations:** Department of Molecular Virology, Graduate School of Medical and Dental Sciences, Tokyo Medical and Dental University, Tokyo, 113-8510, Japan

## Abstract

The growing demands for gene therapy have encouraged development of safe and efficient lentiviral vector (LV) preparation. While much progress has been made in this field, it remains to be explored how to boost its production from producer cells. This paper reports that transient co-expression of RelA or several mitogen-activated protein kinase kinase kinases (MAP3Ks) with packaging constructs can potently enhance LV production in HEK293T producer cells. Adding in transfection a small amount of effector plasmid is sufficient to achieve 3- to 4-fold enhancement, which can further be augmented by co-expression of IκB kinase 2 or HIV Tat. It is also shown that expression of RelA or MAP3K1 can increase LV production in HEK293T/17SF cells grown in suspension. These results indicate that stimulation of intracellular signaling pathways in producer cells represents a powerful means for enhancing LV production.

## Highlights

• RelA increases production of lentiviral vector.
• MAPKKKs increase production of lentiviral vector.
• Tat further enhances lentiviral production by RelA or MAPKKKs.
• RelA and MAPKKK1 increase lentiviral production in suspension cells.

## 1 Introduction

Lentiviral vectors (LVs) have proven safe and useful tools for gene therapy, but the methods for LV production need to be extensively upgraded to meet the currently growing demands [[1], [2], [3]]. One of the big challenges encountered is the difficulty in generating high-titer LV from producer cells. LV can be produced typically by transient transfection of 293 T cell variants in adherent or suspension conditions or by stable producer cell lines. In case of transient transfection, the total amount of plasmid DNAs is limited so that it is required to develop methods that maximize gene expression from the transfected plasmids.

Our previous report demonstrated that a small amount of the Tax protein of human T-cell leukemia type 1 robustly enhances LV production [4]. Tax is a multi-functional viral protein that persistently activates a variety of transcription factors including NF-κB, CREB/ATF, AP-1 and SRF [5]. This led to examining which transcription factors are responsible for Tax enhancement of LV production. Pilot studies thereafter suggested NF-κB and AP-1 as candidates that may play a role therein. RelA is a representative and powerful NF-κB family protein that is normally sequestered in the cytoplasm by inhibitory proteins called IκBs [6]. Upon various stimuli, several MAPK family proteins activate IκB kinases (IKKs), which in turn phosphorylate IκBs and induce their degradation, leading to RelA translocation to the nucleus where it activates target genes. One remarkable feature of these events is that RelA activation terminates shortly because RelA strongly induces expression of IκB proteins which sequester RelA in the cytoplasm unless IKKs are activated again. The AP-1 transcription factor is comprised of the Fos, Jun and ATF family proteins that are mainly activated by JNK1/2/3 and p38 MAPKs [7]. Similar to the NF-κB family proteins, AP-1 activation is tightly regulated by signal transductions through the MAPK pathways. The MAPK signaling cascades consist of sequentially phosphorylated protein kinases called MAP3Ks, MAP2Ks and MAPK that regulate multiple transcription factors including NF-κB and AP-1 simultaneously, resulting in biological processes such as inflammation, proliferation, differentiation and cell death [8]. Each of the complex modes of successive activation of MAP3K, MAP2K and MAP1K leads to changes in expression of a particular set of genes and biological responses.

Consequently, the current work asked if RelA and MAP3Ks can efficiently enhance LV production because MAP3Ks are located upstream of the MAPK signaling cascades and can activate diverse transcription factors.

## 2 Materials and methods

### 2.1 Plasmids

pCSII-CMV-luc-IRES2-Bsd was previously described [4]. pCSII-EF1α-eGFP-IRES-Puro, pCAG-HIVgp and pCMV-VSV-G-RSV-Rev were from RIKEN (RDB12868, RDB04394, RDB04393). pR/cCMVrelA [9], pR/cCMV-IκBαSR encoding a super repressor form of IκBα (Ser32Ala, Ser36Ala) [10], pSV2tat72 [11] and pcDNA3HA [12] were previously described. The cDNA encoding MAP3K1CD was generated by PCR using primers AACCGGATCCGCGTCTCAGGATGCCCTCCCCA and AACCCTCGAGCTACCATGTAGTACGAAAGACT that amplify cDNA encoding the catalytic domain of MAP3K1 (MAP3K1CD). cDNAs encoding MAP3K1CD, MAP3K7 (TAK1) and MAP3K11 (MLK3) were subcloned in pcDNA3HA, and MAP3K8 (TPL2) cDNA was subcloned in pSG5 (Stratagene). The cDNA encoding IKK2EE was generated by PCR using IKK2 cDNA as a template and primers GGCGAACTTTGCACAGAATTCGTGGGGACCCTGCAGTA and GAATTCTGTGCAAAGTTCGCCCTGATCCAGCTCCTTGG for mutagenesis that substitutes glutamate for serine at the amino acid positions 177 and 181. IKK2EE cDNA was then subcloned in pcDNA3 (Invitrogen).

### 2.2 Cells and preparation of the lentiviral vector

Lentiviral vectors were produced by transient transfection of HEK293T cells plated at a density of 3 × 10^5^ cells per collagen-coated well in a 24-well culture plate (VIOLAMO) in 0.5 mL of DMEM (nacalai) supplemented with 10% fetal calf serum (FCS) (Cell Culture Bioscience) and 1% penicillin-streptomycin (nacalai). Cells were maintained for 3 h at 37 °C in a humidified atmosphere with 5 % CO_2_. Cells were then transfected with 0.45 μg of transfer vector, either pCSII-CMV-luc-IRES2-Bsd [4] or pCSII-EF1α-eGFP-IRES-Puro (RIKEN RDB12868), along with packaging plasmids, 0.3 μg of pCAG-HIVgp and 0.2 μg of pCMV-VSV-G-RSV-Rev per well (LV mix), using PEI MAX (Polysciences). Culture medium was replaced with 0.7 mL of fresh medium 24 h after transfection and the viral supernatant was harvested at 48 h after transfection, which was then passed through a filter with a 0.45-μm pore size. HEK293T/17SF cells (ATCC ACS-4500) were purchased from ATCC and cultured in Erlenmeyer flask (VIOLAMO) in BalanCD HEK293 (Irvine Scientific cat#91,165) medium supplemented with 8 mM l-glutamine and 10 μL of insulin-transferrin-selenium (Thermo Fisher). Approximately 1 × 10^6^ HEK293T/17SF cells were aliquoted in 0.5 mL of the medium per well in a 96-well deep well plate (AXYGEN) and transfected with 0.45 μg of pCSII-CMV-luc-IRES2-Bsd, 0.3 μg of pCAG-HIVgp and 0.2 μg of pCMV-VSV-G-RSV-Rev using LV-MAX transfection reagent (Thermo Fisher) according to the manufacturer's protocol. Viral supernatant was harvested at 48 h after transfection. MT4 is a human T-cell line [13] cultured in RPMI (nacalai) supplemented with 10% FCS and 1% penicillin-streptomycin.

### 2.3 Luciferase assay

Approximately 1 × 10^5^ MT4 cells per well in a 96-well plate were plated in 150 μL of RPMI medium supplemented with 10% FCS and 1% penicillin-streptomycin. Cells were incubated with 35 μL of undiluted viral supernatant for 24 h, centrifuged and lysed with 50 μL of luciferase lysis buffer (25 mM Tris pH 7.8, 8 mM MgCl_2_, 1 mM DTT, 1% Triton-X 100, 15% glycerol). Immediately after adding 20 μL of cell lysate to the reaction buffer containing 25 mM Tris pH 7.8, 8 mM MgCl_2_, 1 mM DTT, 15% glycerol, 0.1 mM luciferin (BioThema BT11-500), and 0.5 mM ATP, luciferase activity was measured according to the manufacturer's protocol of the GloMax-Multi Detection system (Promega).

### 2.4 Titration of the lentiviral vector

Approximately 1 × 10^5^ MT4 cells per well in a 96-well plate were plated in 100 μL of RPMI medium supplemented with 10% FCS and 1% penicillin-streptomycin. Lentiviral titers were determined by adding to MT4 cells 10 μL of serially diluted viral supernatant containing the lentiviral vector capable of expressing eGFP. Cells were incubated for 24 h and then supplied with 100 μL of fresh medium. At 48 h after infection, cells were fixed with 1% formalin and the percentage of eGFP-positive cells was determined using flow cytometry.

### 2.5 Western blotting

HEK293T cells 48 h after transfection for lentiviral production were lysed in lysis buffer (25 mM Tris pH 7.8, 8 mM MgCl_2_, 1 mM DTT, 1% Triton-X 100, 15% glycerol). Protein concentration was determined by the Bradford assay. Approximately 30 μg of proteins were separated by SDS-PAGE, transferred to polyvinylidene difluoride (PVDF) membranes and reacted with a rat monoclonal antibody to the influenza virus haemagglutinin (HA) (3F10, Roche Applied Science), rabbit polyclonal antibody to RelA (C-20 SantaCruz), rabbit monoclonal antibody to TPL2 (71184S Cell signaling) or mouse monoclonal antibody to GAPDH (GT239, GeneTex). Membranes were then incubated with horseradish peroxidase-conjugated goat anti-rat IgG (sc-2006, Santa Cruz) or anti-mouse IgG (MerckMillipore) and proteins were visualized by western Lightning Plus-ECL or Western Lightning Ultra (PerkinElmer) and ODYSSEY® Fc Imaging System.

## 3 Results

### 3.1 RelA and IKK2EE enhance lentiviral production in HEK293T cells

**Fig. 1:**
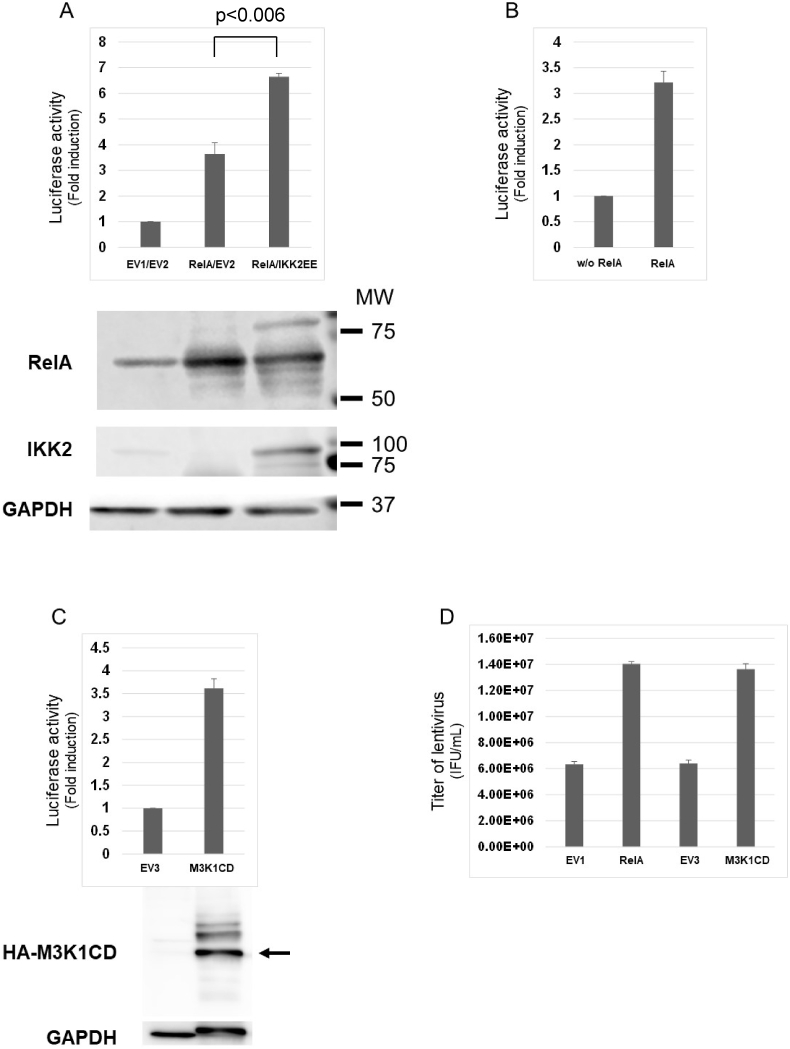
(A) Approximately 3 × 10^5^ HEK293T cells in a 24-well plate were transfected with 0.45 μg of pCSII-CMV-luc-IRES2-Bsd, 0.3 μg of pCAG-HIVgp and 0.2 μg of pCMV-VSV-G-RSV-Rev (LV mix) together with 0.1 μg of pR/cCMV (EV1) or pR/cCMVrelA (RelA) and 0.025 μg of pcDNA3 (EV2) or pcDNA3IKK2EE (IKK2EE). At 48 h after transfection, approximately 1 × 10^5^ MT4 cells were incubated with 35 μL of viral supernatant for 24 h, lysed and subjected to luciferase assay. The results are shown as fold-increase compared to the control. The bars indicate standard errors calculated from three independent experiments. Transfected HEK293T cells were lysed with luciferase lysis buffer at 48 h after transfection. Approximately 30 μg of proteins was subjected to western blotting with anti-RelA, anti-IKK2 or anti-GAPDH antibodies. (B) Approximately 7.2 × 10^6^ HEK293T cells in a 10 cm dish were transfected with 10.8 μg of pCSII-CMV-luc-IRES2-Bsd, 7.2 μg of pCAG-HIVgp and 4.8 μg of pCMV-VSV-G-RSV-Rev (LV mix) with or without 1.09 μg of pR/cCMVrelA (RelA). Luciferase activity was measured 48 h after infection of MT4 cells. The bars indicate standard errors calculated from three independent experiments. (C) HEK293T cells were transfected as in (A) with LV mix together with 0.05 μg of pcDNA3HA (EV3) or pcDNA3HA-MAP3K1CD (MAP3K1CD). Luciferase activity was measured as in (A). Approximately 30 μg of proteins was subjected to western blotting with anti-HA or anti-GAPDH antibodies. The arrow indicates the position of HA-tagged MAP3K1CD. (D) LV titers were determined by measuring percentages of eGFP-positive cells. The results are shown as average of IFU/mL calculated from three independent experiments. The bars indicate standard errors.

A transfer vector pCSII-CMV-luc-IRES2-Bsd capable of expressing firefly luciferase reporter gene was used because luciferase activity in target cells is supposed to represent the yield of functional lentivirus vector in the culture medium. The transfer vector and packaging constructs used in this study carry the cytomegalovirus (CMV) immediate early enhancer, which has multiple transcription factor binding sites including Nuclear Factor κB (NF-κB), Activator Protein-1 (AP-1) and Serum Response Factor (SRF) [14]. Thus, it was first examined if RelA, a representative NF-κB transcription factor that is normally sequestered in the cytoplasm by an inhibitory protein IκBα but can activate target genes if transiently overexpressed, enhances lentiviral production. As shown in Fig. 1A, addition of only 0.1 μg of the RelA-expression plasmid, nearly the one-tenth amount of the plasmid for usual lentiviral production in a 24-well, was sufficient to achieve 3.5-times more luciferase activity in target MT4 cells. This increase in luciferase activity was further potentiated by co-expression of IKK2EE (p < 0.006), a constitutively active form of IκBα kinase 2 (IKK2) [15], which phosphorylates and induces degradation of IκBα bound to RelA in the cytoplasm (Fig. 1A). Expression of each effector protein in producer cells was verified by western blotting. In addition, RelA enhanced LV production in a larger-scale culture condition as well (Fig. 1B).

### 3.2 MAP3Ks enhance lentiviral production in HEK293T cells

In line with an idea that simultaneous activation of cellular transcription factors may lead to stronger activation of the CMV immediate early enhancer present in transfer and packaging plasmids, MAP3Ks that potentially activate multiple transcription factors were considered as top-priority candidates for examination. The catalytic domain of MAP3K1 lacking its amino-terminal regulatory domains (MAP3K1CD) was firstly examined because this kinase is usually under tight negative feedback control through the regulatory domains [16]. As expected, MAP3K1CD strongly enhanced LV production as shown by the luciferase activity (Fig. 1C). Expression of HA-tagged MAP3K1CD in producer cells is shown at the bottom. The band for GAPDH appears shifted upward in the presence of exogenous MAP3K1CD most likely due to direct or indirect phosphorylation by its aberrant catalytic activity. The titer of LV measured using pCSII-EF1α-eGFP-IRES-Puro capable of expressing eGFP in infected cells reached the 10^7^ TU/mL range by transient co-expression of RelA or MAP3K1CD, suggesting the validity of this experimental system (Fig. 1D). Moreover, combined expression of RelA and MAP3K1CD achieved further enhancement (Supplementary Fig. 1A). Since MAP3K1 reportedly activates NF-κB reporter gene expression when transiently expressed [17], it is possible that MAP3K1CD enhances LV production through NF-κB activation. To test this possibility, RelA or MAP3K1CD were expressed in the presence of a super repressor form of IκBα (SR) that cannot be phosphorylated by IκB kinases due to amino acid substitutions of Alanine for Serine at the amino acid positions 32 and 36 [10]. Of note, co-expression of SR reversed the enhancement by ReA while SR didn't that by MAP3K1CD, suggesting that MAP3K1CD works on LV production separately from NF-κB signaling (Supplementary Figs. 1B and 1C).

**Fig. 2:**
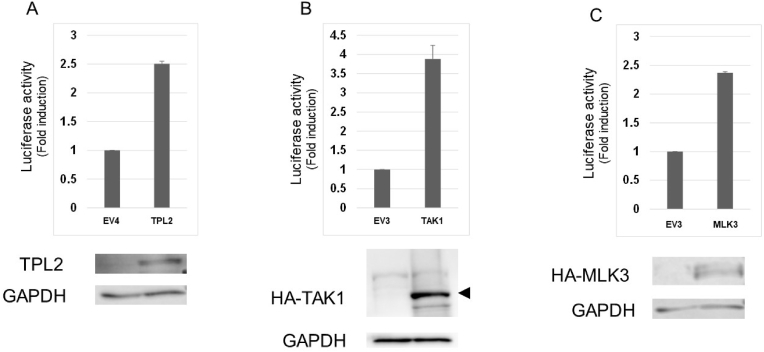
(A) HEK293T cells were transfected with LV mix together with 0.15 μg of pSG5 (EV4) or pSG5-TPL2 (TPL2). Luciferase activity was measured and shown as in Fig. 1 (A). Approximately 30 μg of proteins was subjected to western blotting with anti-TPL2 or anti-GAPDH antibodies. (B) HEK293T cells were transfected with LV mix together with 0.05 μg of pcDNA3HA (EV3) or pcDAN3HA-TAK1 (TAK1). Luciferase activity was measured and shown as in Fig. 1 (A). Approximately 30 μg of proteins was subjected to western blotting with anti-HA or anti-GAPDH antibodies. (C) HEK293T cells were transfected with LV mix together with 0.025 μg of pcDNA3HA (EV3) or pcDAN3HA-MLK3 (MLK3). Luciferase activity was measured and shown as in Fig. 1 (A). Approximately 30 μg of proteins was subjected to western blotting with anti-HA or anti-GAPDH antibodies.

**Fig. 3:**
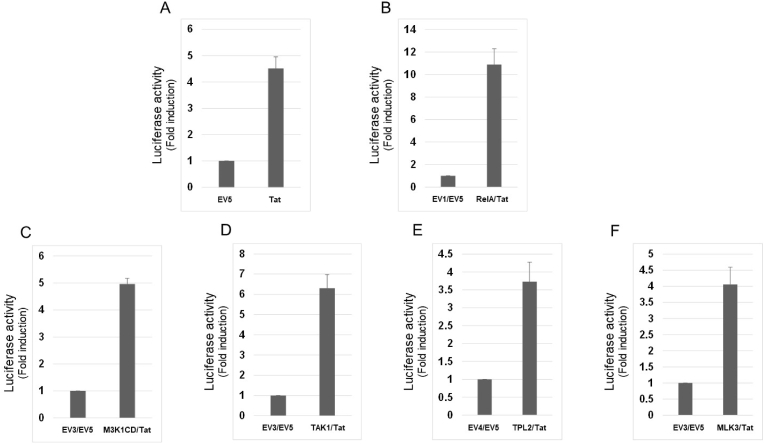
(A) HEK293T cells were transfected as with LV mix together with 0.1 μg of pSV2 (EV5) or pSV2tat72 (Tat). Luciferase activity was measured and shown as in Fig. 1 (A). (B) HEK293T cells were transfected with LV mix together with 0.1 μg of EV1 and 0.1 μg of EV5 (left) or 0.1 μg of pR/cCMVrelA (RelA) and 0.1 μg of pSV2tat72 (Tat) (right). Luciferase activity was measured and shown as in Fig. 1 (A). (C) HEK293T cells were transfected with LV mix together with 0.025 μg of EV3 and 0.1 μg of EV5 (left) or 0.1 μg of pcDNA3HA-MAP3K1CD (M3K1CD) and 0.1 μg of pSV2tat72 (Tat) (right). Luciferase activity was measured and shown as in Fig. 1 (A). (D) HEK293T cells were transfected with LV mix together with 0.05 μg of EV3 and 0.1 μg of EV5 (left) or 0.1 μg of pcDNA3HA-TAK1 (TAK1) and 0.1 μg of pSV2tat72 (Tat) (right). Luciferase activity was measured and shown as in Fig. 1 (A). (E) HEK293T cells were transfected with LV mix together with 0.15 μg of EV4 and 0.1 μg of EV5 (left) or 0.15 μg of pSG5-TPL2 (TPL2) and 0.1 μg of pSV2tat72 (Tat) (right). Luciferase activity was measured and shown as in Fig. 1 (A). (F) HEK293T cells were transfected with LV mix together with 0.025 μg of EV3 and 0.1 μg of EV5 (left) or 0.1 μg of pcDNA3HA-MLK3 (MLK3) and 0.1 μg of pSV2tat72 (Tat) (right). Luciferase activity was measured and shown as in Fig. 1 (A).

Among other MAP3Ks, MAP3K7 (TAK1) [18], MAP3K8 (TPL2) [19] and MAP3K11 (MLK3) [20] were found to boost LV production effectively in HEK293T cells (Fig. 2 A, 2B and 2C). Expression of each effector protein in producer cells was verified by western blotting. Moreover, Tat, the HIV-derived transcription elongation factor [11], not only enhances LV production by itself, but also cooperates with these effectors in LV production when co-expressed in producer cells using an independent expression plasmid (Fig. 3 A to F).

### 3.3 RelA and MAP3K1CD enhance LV production in suspension cells

**Fig. 4:**
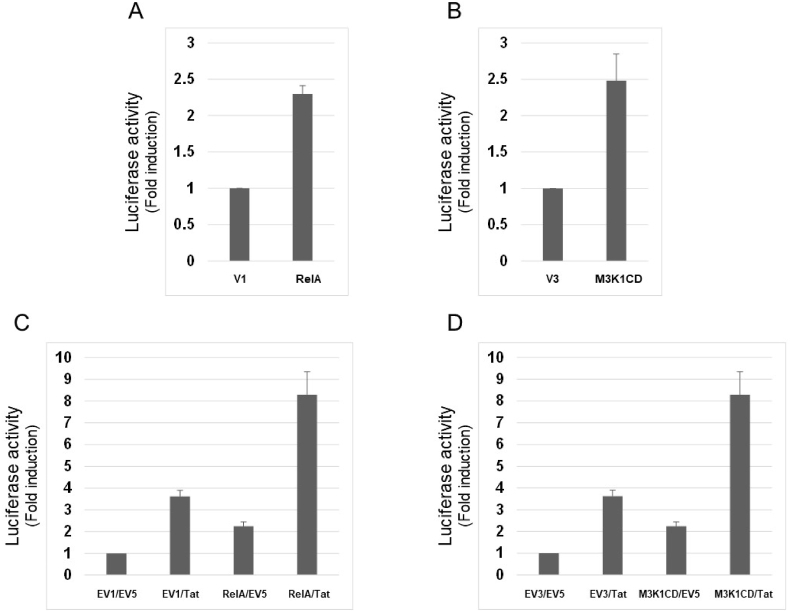
(A) Approximately 1 × 10^6^ HEK293T/17SF cells were transfected with 0.45 μg of pCSII-CMV-luc-IRES2-Bsd, 0.3 μg of pCAG-HIVgp and 0.2 μg of pCMV-VSV-G-RSV-Rev (LV mix) together with 0.3 μg of pR/cCMV (EV1) or pR/cCMVrelA (RelA). Luciferase activity was measured and shown as in Fig. 1 (A). (B) HEK293T/17SF cells were transfected with LV mix together with 0.2 μg of EV3 or 0.2 μg of pcDNA3HA-MAP3K1CD (M3K1CD). Luciferase activity was measured and shown as in Fig. 1 (A). (C) HEK293T/17SF cells were transfected with LV mix together with effector plasmids in 4 different combinations: 0.3 μg of EV1 and 0.2 μg of EV5; 0.3 μg of EV1 and 0.2 μg of pSV2tat72; 0.3 μg of pR/cCMVrelA and 0.2 μg of EV5; 0.3 μg of pR/cCMVrelA and 0.2 μg of pSV2tat72. Luciferase activity was measured and shown as in Fig. 1 (A). (D) HEK293T/17SF cells were transfected with LV mix together with effector plasmids in 4 different combinations: 0.3 μg of EV3 and 0.2 μg of EV5; 0.3 μg of EV3 and 0.2 μg of pSV2tat72; 0.3 μg of pcDNA3HA-MAP3K1CD and 0.2 μg of EV5; 0.3 μg of pcDNA3HA-MAP3K1CD and 0.2 μg of pSV2tat72. Luciferase activity was measured and shown as in Fig. 1 (A).

Finally, it was examined if RelA and MAP3K1CD can potentiate LV production in suspension cells because such producer cells are considered more suitable for large-scale production of LVs for gene therapy. 293T/17SF cells were transfected with the same set of LV constructs as 293 T cells were, and viral supernatants were recovered 48 h after transfection for infection of MT4 cells. Adding expression plasmid for RelA or MAP3K1CD to the usual transfection mix elevated luciferase activity by two-to three-fold (Fig. 4). This enhancement was further potentiated by Tat co-expression as in adherent 293 T cells.

## 4 Discussion

Many factors such as the type of cell line, culture conditions including the bioreactor, media, cell densities and the methods of transfection have been focused and investigated as important factors to improve LV production, but it remains to be developed how to boost LV production by managing gene expression in producer cells. An idea that underlies this study is that changes in cellular gene expression, not necessarily limited to that from the transfected plasmids, could enhance LV production in producer cells. As an effective means to alter a broad spectrum of gene expression, the activity of transcription factors was managed by expressing a transcription factor itself or cellular kinases like MAP3Ks that can impact on multiple transcription factors. The presence of many transcription factor-binding sites in the CMV immediate early enhancer suggests that the effectors used in this study enhanced gene expression from the transfer and packaging constructs as we previously demonstrated for the Tax protein of HTLV-I [4]. Indeed, our preliminary transient transfection experiments indicated that RelA and MAP3K1CD strongly activate the CMV promoter (data not shown), but it is reasonable to assume that the effectors also changed expression of cellular genes important for the viability of producer cells or involved in the processes of LV virion formation and release.

Tax strongly enhanced LV production, activating a variety of transcription factors, but its oncogenicity may not be acceptable for production of LVs used in gene therapy [21]. One notable host transcription factor persistently activated by Tax is NF-κB, and as shown above, transient co-expression of RelA, which has not been directly implicated in oncogenesis, efficiently boosted LV production. Another major transcription factor targeted by Tax is AP-1 comprised of the Fos, Jun and ATF family proteins, whose activity is mainly regulated by the host JNK1/2/3 and p38 MAPK1 proteins [7]. Accordingly, this study sought to find host kinases that are mapped upstream of, and can concomitantly activate, JNK1/2/3 and/or p38 MAPK1s, and discovered here that MAP3K1CD, TAK1, TPL2 and MLK3 are capable of enhancing LV production. The precise mechanisms of how these MAP3Ks work on LV production remain to be studied due to the complexity of actions of these MAP3Ks in multiple signal transduction pathways [8]. For example, MAP3K1CD indeed activates NF-κB reporter gene expression [17], but the result that SR does not impede enhanced LV production by MAP3K1CD suggests that transcription factors other than NF-κB contribute to enhancing LV production. Another example is the cooperation of TPL2 and MLK3 in LV production, which may result from their different modes of actions on LV production.

It was shown here that Tat, when expressed *in trans* from an independent plasmid, can further enhance LV production. It has been considered that the third generation of packaging system employing a strong enhancer in transfer vector does not require Tat for high titer LV production [22, 23]. Expression of vector RNA is one of the important rate-limiting steps in LV production, which is controlled in part by transcriptional elongation through the trans-acting responsive element (TAR) element present in the R region of HIV long terminal repeat (LTR). This elongation of transcripts is an event independent from the initiation of transcription which can markedly be promoted by exogenous enhancers such as that derived from CMV. Thus, it is not surprising that Tat co-operated to enhance LV production under the control of the CMV enhancer.

Finally, the results that RelA and MAP3K1CD enhanced LV production in 293T/17SF cells grown in suspension suggest that this method can be applicable to a large-scale LV production both in academic and industrial sectors.

## CRediT authorship contribution statement

**Shoji Yamaoka:** Writing – original draft, Resources, Project administration, Methodology, Investigation, Funding acquisition, Formal analysis, Data curation, Conceptualization.

## Declaration of competing interest

The author has no known competing financial interests or personal relationships that might affect the research reported in this paper.
