# Supplementary material for: RelA and mitogen-activated protein kinase kinase kinases potently enhance lentiviral vector production
Source: Biochem Biophys Rep. 2024 Feb 1;37:101637. doi: 10.1016/j.bbrep.2024.101637 (PMC10847020; doi:10.1016/j.bbrep.2024.101637)
Supplement: Multimedia component 1 [file mmc1.pptx]

## Slide 1
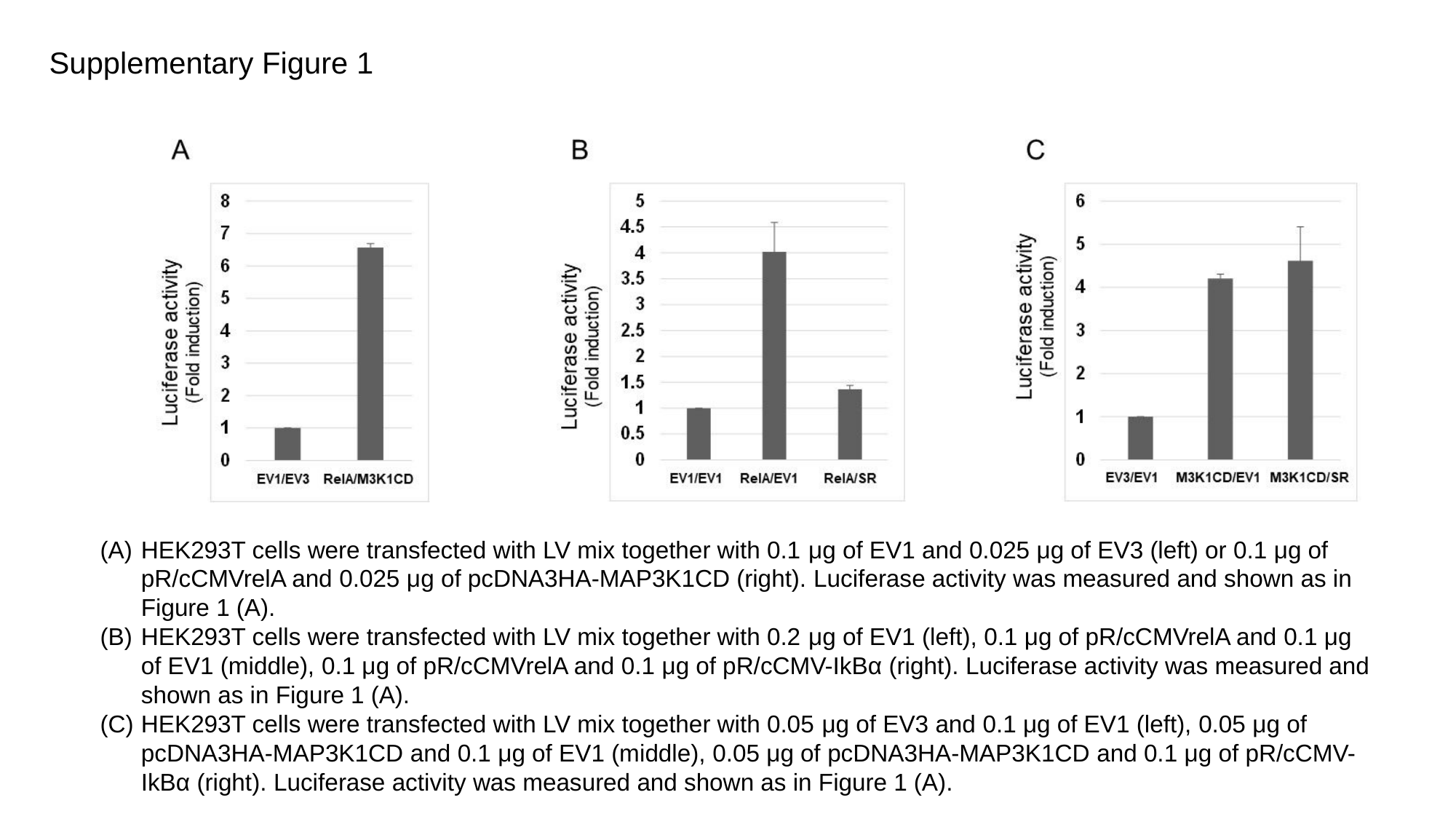

Supplementary Figure 1
HEK293T cells were transfected with LV mix together with 0.1 μg of EV1 and 0.025 μg of EV3 (left) or 0.1 μg of pR/cCMVrelA and 0.025 μg of pcDNA3HA-MAP3K1CD (right). Luciferase activity was measured and shown as in Figure 1 (A).
HEK293T cells were transfected with LV mix together with 0.2 μg of EV1 (left), 0.1 μg of pR/cCMVrelA and 0.1 μg of EV1 (middle), 0.1 μg of pR/cCMVrelA and 0.1 μg of pR/cCMV-IkBα (right). Luciferase activity was measured and shown as in Figure 1 (A).
HEK293T cells were transfected with LV mix together with 0.05 μg of EV3 and 0.1 μg of EV1 (left), 0.05 μg of pcDNA3HA-MAP3K1CD and 0.1 μg of EV1 (middle), 0.05 μg of pcDNA3HA-MAP3K1CD and 0.1 μg of pR/cCMV-IkBα (right). Luciferase activity was measured and shown as in Figure 1 (A).
